# What would I do? Perspectives on the factors underlying Lynch syndrome genetic testing and results sharing decisions for high‐risk colorectal cancer patients

**DOI:** 10.1002/pon.5840

**Published:** 2021-11-08

**Authors:** Gabriella Tiernan, Victoria Freeman, April Morrow, Emily Hogden, Karen Canfell, Yoon‐Jung Kang, Natalie Taylor

**Affiliations:** ^1^ The Daffodil Centre The University of Sydney, a joint venture with Cancer Council NSW Sydney New South Wales Australia; ^2^ Prince of Wales Clinical School University of New South Wales Sydney New South Wales Australia; ^3^ School of Population Health, Faculty of Medicine University of New South Wales Sydney New South Wales Australia

**Keywords:** attitudes, colorectal neoplasms, decision making, genetic testing, health knowledge, hereditary nonpolyposis, practice, psycho‐oncology, qualitative research

## Abstract

**Objective:**

Universal tumour testing for Lynch syndrome (LS) in all incident colorectal cancers (CRCs) and sequential diagnostic genetic testing is cost‐effective in Australia. Because of this, our study aimed to understand factors underlying possible decisions faced by tumour test‐positive CRC patients and their at‐risk relatives throughout the LS diagnosis pathway.

**Methods:**

Semi‐structured telephone interviews were conducted with 23 participants, using four hypothetical scenarios. Vignette‐guided closed‐ and open‐ended questions asked about LS genetic testing uptake, discussing diagnosis with at‐risk relatives, and risk‐reducing interventions. Personal perspectives on genetic testing were collected pre‐post vignette discussion. Inductive thematic analysis was performed on open‐ended questions. Decisional pathway diagrams were developed to convey factors influencing complex decision‐making processes.

**Results:**

Participant responses incorporated unfolding scenario information, resulting in three decision themes: (1) wanting to know one's LS status; (2) informing family about LS; (3) navigating risk‐reducing interventions. Across all themes, ‘knowledge’ emerged as a facilitator, and ‘negative emotional experience’ as a barrier. Personal supportive views toward genetic testing increased post‐interview.

**Conclusions:**

When communicating with tumour test‐positive CRC patients or their relatives about LS genetic testing, providing guidance/resources to inform decisions around risk‐reducing interventions and informing family members is critical. Scenario‐driven interviews provide insight into what individuals might do when facing complex healthcare decisions and could aid informed decision‐making. This approach may be applicable in other conditions, particularly with mainstreaming being increasingly introduced into the genetic context.

## 1 INTRODUCTION

Lynch syndrome (LS) is a hereditary cancer‐susceptibility disorder associated with an increased risk of developing a range of cancers, particularly colorectal cancer (CRC).^1^ Universal tumour testing in all incident CRC cases to identify tumours with DNA mismatch deficiencies (dMMR) followed by sequential diagnostic germline genetic testing to confirm LS is cost‐effective in Australia and other developed countries.^2^, ^3^ It's cost‐effectiveness improves if more mutation carriers (i.e. probands) undertake diagnostic genetic testing and communicate results with at‐risk family members, so family cascade testing rates increase.^2^ This approach is likely to be more systematically implemented in Australia.^4^ Universal LS genetic testing in all CRC cases is not currently cost‐effective, but may eventually become cost‐effective should costs decrease.

CRC patients with dMMR tumours have a high chance (up to 67%) of carrying an underlying germline LS mutation.^5^, ^6^ If patients are found to be probands, their at‐risk relatives can undergo predictive genetic testing to clarify their own cancer risks^7^, ^8^ (further details in Supporting Information). Even if a germline LS mutation is not detected despite dMMR (“Lynch‐like syndrome”), patients may still have elevated cancer risks and may benefit from personalised surveillance plans.^9^ However, Australian evidence has found that only around half of tumour‐test positive cases choose to have genetic testing.^10^ Therefore, it is important to understand factors affecting decision‐making processes regarding LS genetic testing for individuals diagnosed with tumour‐test positive CRC, and if LS confirmed, their at‐risk relatives.

These individuals must consider possible consequences, including increased cancer risks to themselves and at‐risk relatives (e.g., children, siblings), risk‐reducing interventions (e.g., colonoscopic surveillance, surgery), and life insurance implications.^11^, ^12^, ^13^ To provide effective decision‐making support to affected individuals, healthcare providers must understand facilitators and barriers associated with these decision‐making processes. To date, Australian studies have restricted decision‐making research to those already commencing LS testing and/or are confirmed carriers.^10^, ^14^, ^15^, ^16^, ^17^, ^18^ CRC is the second most common cancer in Australia,^19^ and universal genetic testing for LS on CRC may eventually become cost‐effective.^2^ Therefore understanding perspectives around genetic testing and subsequent decision‐making processes is important.

This study aimed to understand perspectives towards LS genetic testing for newly diagnosed tumour‐positive CRC patients and relevant events following LS diagnosis. Specific aims were to identify and explore: (i) key considerations and decision‐making processes when facing hypothetical scenarios about LS testing; and (ii) facilitators and barriers associated with key considerations identified.

## 2 METHODS

Semi‐structured interviews were conducted around evolving hypothetical scenarios (vignettes) describing tumour test‐positive CRC and subsequent LS diagnosis and related events (further details in Supporting Information). Quantitative analysis was performed on close‐ended questions and inductive thematic analysis on open‐ended questions to identify key themes and subthemes. Decisional pathway diagrams captured decision‐making processes, and facilitators or barriers encountered. Conduct and reporting of this study complies with the consolidated criteria for reporting qualitative studies (COREQ) checklist^20^ (Table S1).

### 2.1 Participant recruitment

Following ethical approval from Cancer Council NSW (CCNSW) Human Research Ethics Committee (#316), individuals attending an in‐person, large‐scale Australian cancer charity fundraising event in May 2018 were invited to participate in a telephone interview. Those interested were given a study verbal outline and consent form. Consenting participants were given an LS factsheet and interview vignettes (further details in Supporting Information). Inclusion criteria were: no current or previous cancer diagnoses, aged 18 years or over, and ability to communicate in spoken English.

### 2.2 Interview schedule development and data collection

Vignettes about a family affected by LS were developed through consulting representatives from consumer organisation Lynch Syndrome Australia (LSA). Vignettes follow a proband ‘James’ and sister ‘Sarah’ throughout stages of the LS diagnostic pathway. Scenarios included LS genetic testing uptake following tumour test‐positive CRC diagnosis, discussing subsequent LS diagnosis with at‐risk relatives, considering risk‐reducing interventions, and involving healthcare professional support. The interview schedule was piloted and refined.

In one‐on‐one telephone calls (conducted by Yoon‐Jung Kang, April Morrow, and Victoria Freeman), participants were first asked for personal opinions about genetic testing and given a verbal summary of the LS factsheet. They were presented with the four‐part evolving scenario, each followed by closed‐ and open‐ended questions, and asked to answer from the protagonist's perspective. At conclusion, participants were asked if personal views about genetic testing had changed. Interviews were audio recorded, transcribed verbatim, and checked for accuracy (Victoria Freeman, Gabriella Tiernan).

### 2.3 Data analysis

#### 2.3.1 Quantitative analysis

Responses to five close‐ended questions were independently categorised (Victoria Freeman, Gabriella Tiernan) then discussed to achieve consistency.

#### 2.3.2 Thematic analysis

All transcripts were de‐identified and imported into NVivo 12 Plus software (QSR International Pty Ltd 2018). An inductive thematic analytic approach was taken by two researchers (Gabriella Tiernan, Victoria Freeman).^21^ Transcripts were initially reviewed independently without coding and preliminary notes taken. A blinded iterative complete coding approach was taken.^22^ Coding structures were shared, discrepancies discussed, and a common structure was formed. This was applied to re‐code all transcripts. Similar codes were grouped to develop and refine key themes and subthemes based on quotation salience and volume. A third independent reviewer resolved discordances (Natalie Taylor). Decision pathway diagrams were developed to convey factors influencing decision‐making processes around key themes. During analysis write‐up, key themes or subthemes were further refined (Gabriella Tiernan, Victoria Freeman) with input from the research team (Natalie Taylor, Yoon‐Jung Kang, April Morrow).

## 3 RESULTS

Forty‐four individuals consented to participate, and 18 completed the telephone interview (38% response rate). Twenty‐six individuals either withdrew (*n* = 3), or did not respond (*n* = 23). To reach optimal sample size,^23^ we recruited a convenience sample until data reached saturation, resulting in five additional participants (participant numbers: 45, 46, 47, 48, 49) and totalling 23 participants (Table S1 for further details).

Three key themes and accompanying subthemes emerged from open‐ended responses: (a) Wanting to know of one's LS status (subthemes: knowledge is power, fear of testing positive, personal beliefs); (b) Informing family about LS (subthemes: responsibility, support, privacy); (c) Navigating risk‐reducing interventions (subthemes: prevention of cancer, physical implications, benefit outweighs risk). Each theme's findings are presented at the following levels:

1. Theme: represents a key decision involved in processing a LS diagnosis.
2. Initial considerations: factors initially considered within the decision.
3. Theme‐based barriers and facilitators: factors discouraging or encouraging participants toward a decisional outcome (may include factors also identified as subthemes).
4. Positive reinforcers and enablers: Positive reinforcers were factors which could strengthen a facilitator in relation to a decisional outcome, and enablers were factors which may weaken a barrier in relation to a decisional outcome.
5. Negative reinforcers: factors which could strengthen a barrier in relation to a decisional outcome.
6. Subthemes: represents the dominant factors considered within decision‐making processes.
7. Decisional outcome(s): summary of participants' intended behaviours on the theme.

Table S2 contains quotes supporting each theme and associated barriers and facilitators (Table S4. For extended quotes). Codes associated with each quote according to theme, and barrier/facilitator are used to support in‐text results (e.g. A.Fr.1 refers to Theme A, facilitator and key first quote listed). Some representative quotes are cited in‐text and labelled by participant number (e.g., P22) or convenience sample number (e.g., CP45).

Results indicated that participants used the genetic testing and LS information from the interview to weigh‐up considerations and inform hypothetical decisions made on behalf of the characters. Complex decision‐making pathways were revealed (Figure 1), and thinking patterns tended to evolve as scenarios unfolded and participants became more informed.

FIGURE 1Decisional pathway diagrams showing barriers and facilitators identified within each theme. (A) Wanting to know of one's LS status; (B) Informing family about LS; and (C) Navigating risk reducing interventions. LS, Lynch syndrome; GT, Genetic testing; HCP, Healthcare professional

**Figure:**
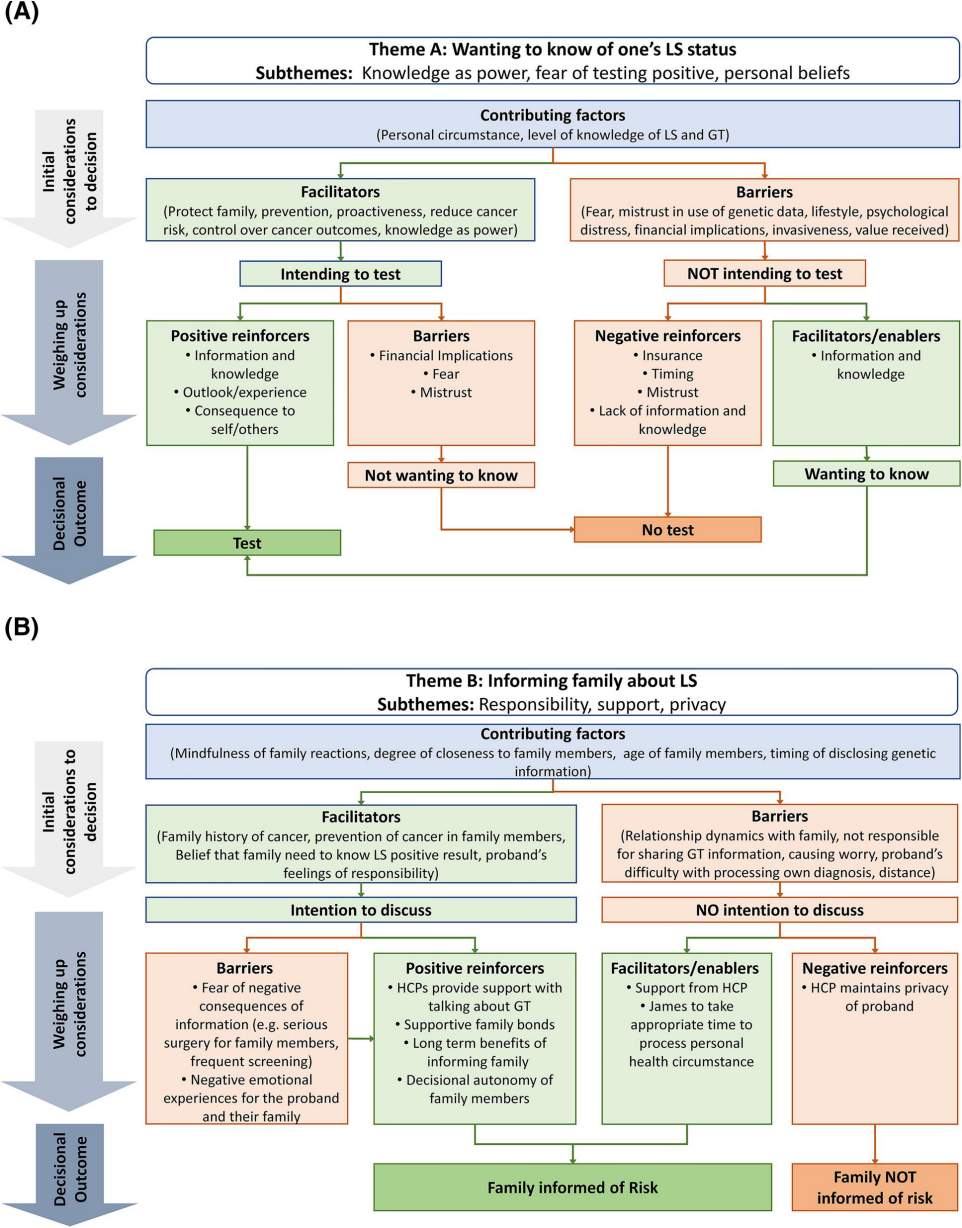

**Figure:**
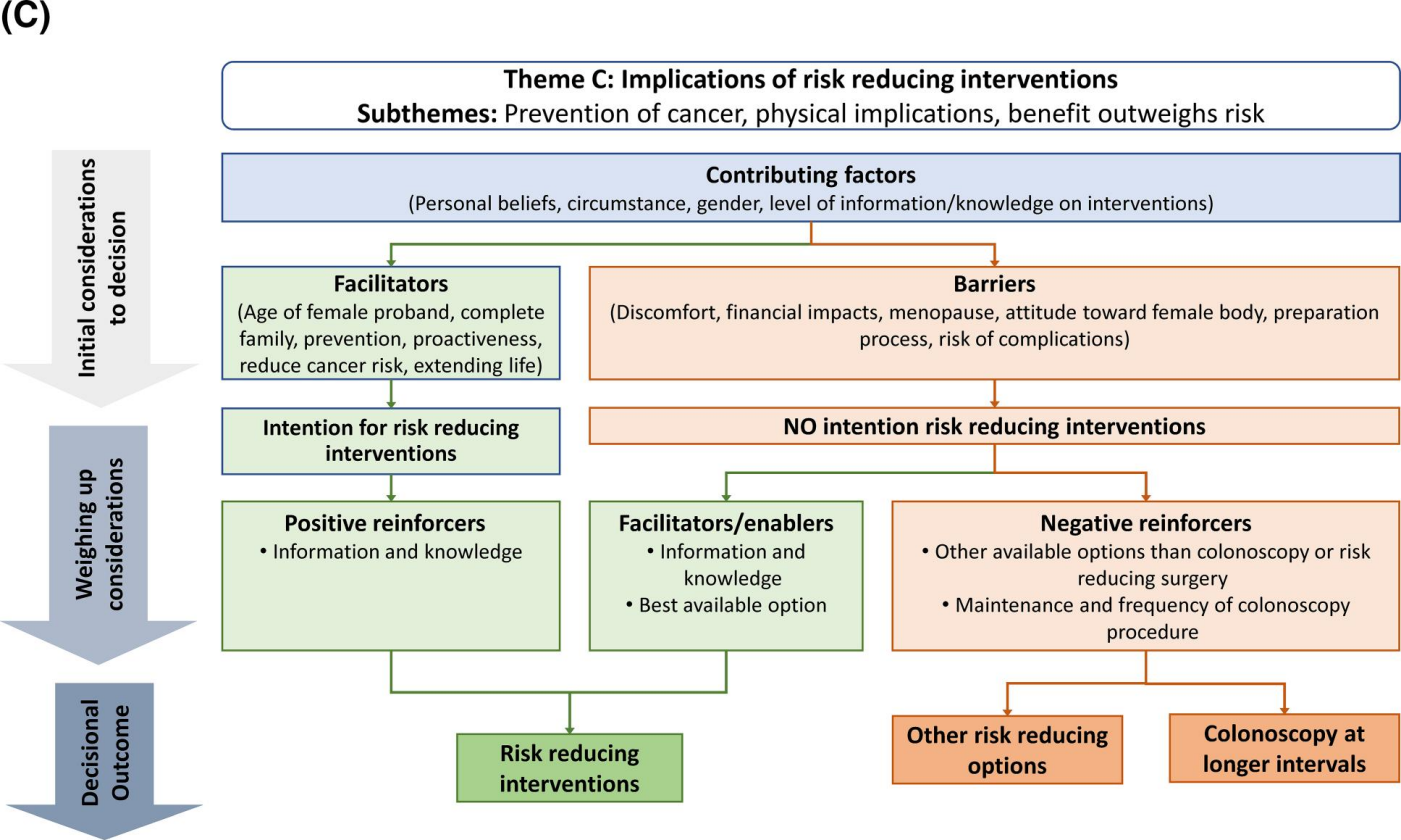

Of participants who initially provided personal opinions about genetic testing (*n* = 20), 60% (*n* = 12) were in favour, which increased to 85% (*n* = 17) at interview conclusion (Table S3). Those initially conditionally in favour [that is, provided specified conditions were met] (*n* = 4), and neutral, open‐minded or unsure (*n* = 4) towards genetic testing, became in favour by conclusion. One participant remained unsure and two participants initially favouring genetic testing changed their views to conditionally in favour of, or unsure about testing. Of the 23 participants asked at interview conclusion, 91% (*n* = 21) were in favour of genetic testing.

### 3.1 Theme A: wanting to know of one's LS status

#### 3.1.1 Theme

Working through the vignettes, participants felt wanting to know of one's LS status was a key factor in this process.

#### 3.1.2 Initial considerations

Personal circumstance, personal beliefs, and knowledge levels about LS and genetic testing were initially raised and may immediately influence the decision to know of one's LS status (Figure 1A).

#### 3.1.3 Associated barriers and facilitators

Barriers included unintended negative consequences (e.g., genetic information misuse or effects upon insurance policies), financial impacts, invasiveness of procedure, the belief that genetic testing may not be necessary (A.Br.1), and fear of testing positive for LS.

Facilitating factors included protecting one's family (A.Fr.1), taking a preventative and proactive approach (A.Fr.2), and controlling one's risk of cancer. Knowledge was a frequently mentioned facilitator, both with regards to having knowledge of one's genetic risk, and having knowledge of genetic testing and LS.

#### 3.1.4 Positive reinforcers and enablers

Knowledge was perceived as a positive reinforcer in deciding to uptake testing as well as enabler which may overcome barriers to testing (e.g., fear and mistrust of procedure) (A.Fr.4).

#### 3.1.5 Negative reinforcers

Consistent with this finding, lack of knowledge was a negative reinforcer (A.Br.4). Participants commonly felt discouraged by the consideration of insurance companies and felt they may take advantage of genetic results (A.Br.5).

#### 3.1.6 Subthemes

“Knowledge is power” was a commonly expressed attitude facilitating the desire to know LS status. Participants described knowing one's genetic risk may enable better management of their cancer risk: *“you've got the power to either do what you want to do to try and not get the cancer…the earlier the detection the better your chances are of survival”* P9.

Personal beliefs on genetic testing were a key influence in deciding to know of one's risk, which may facilitate a decision in favour, or not in favour of knowing one's genetic risk. Despite being informed (via pre‐interview information, which interviewers offered to read aloud) about how genetic factors related to a LS diagnosis may increase cancer risk, one participant felt genetic testing may not be necessary due to beliefs that cancer risk may be controlled by “lifestyle changes” such as diet and exercise (A.Br.2).

Fear of testing positive was another key influence on wanting to know, where most participants acknowledged possible negative events which may follow a positive result. One participant described knowing one's LS status may cause severe psychological distress and possible self‐harm (A.Br.3).

#### 3.1.7 Decisional outcomes

After exploring considerations around deciding to uptake genetic testing, most participants (*n* = 22/23) answered in favour of both James and Sarah undertaking genetic testing and being informed of their LS status (Table S3; Figure 1A).

### 3.2 Theme B: informing family about LS

#### 3.2.1 Theme

Participants felt the decision to inform at‐risk family members about LS was a key consideration in the LS diagnosis scenario.

#### 3.2.2 Initial considerations

Family reactions, degree of closeness to family members, age of family members, and timing of genetic information disclosure were immediately considered, *“I don't think it would be necessary to tell her kids before getting the test…best to get the test and if it comes back positive then tell her kids and any other immediate family…”* CP48 (Figure 1B).

#### 3.2.3 Associated barriers and facilitators

Anxiety and other negative emotions resulting from discussions about LS were identified as barriers to informing family (B.Br.1), (B.Br.2). The proband's difficulty with processing their own diagnosis was also a perceived barrier (B.Br.3). Physical and emotional distance with family members were additional barriers.

Strong family cancer history and possibility of preventing future cancer in family members were facilitators to informing family of LS (B.Fr.1). Family needing to know for their own safety was frequently expressed, *“…you could be endangering their life by not telling them”* P8, and it being the proband's responsibility to inform their family.

#### 3.2.4 Positive reinforcers and enablers

Processing one's diagnosis was identified as an enabler towards a proband informing at‐risk family members about LS.

Healthcare professionals supporting conversations between proband and family was a positive reinforcer and enabler. Support included being present during conversations or preparing the proband through informational and counselling support beforehand (B.Fr.2).

If a proband decided against informing at‐risk family, some participants felt it was the healthcare professional's responsibility to disclose their results to family (enabler).

#### 3.2.5 Negative reinforcers

Patient privacy was a negative reinforcer where some participants felt there was no justification to disclosing LS positive results to family members without the proband's consent, “*I don't see why automatically the doctor should be able to say to the family member ‘Hey, James has got Lynch syndrome’ as opposed to Ebola or something that's highly infectious*” P42.

#### 3.2.6 Subthemes

Support was a key influence in informing family members. Emotional support from family members was viewed as facilitator towards the proband having conversations with other at‐risk relatives (B.Fr.3). Receiving informational and/or counselling support from healthcare professionals was also viewed as a facilitator. Some participants suggested the genetic counsellor could attend family conversations (B.Fr.2).

Perceptions around responsibility strongly influenced deciding to inform at‐risk family members. Most participants (*n* = 21/23) felt the proband held responsibility to inform family members of their LS risk (Table S3). Strong family history of cancer, potential to prevent cancer in family members and believing family members need to know of the proband's LS diagnosis informed this view, “*it affects more than me*” P1. If a proband decided not to share genetic results with at‐risk family members, approximately half (*n* = 11/23) (Table S3) felt the health care professional held responsibility to inform the proband's family (B.Fr.4), but half (*n* = 10/23) disagreed, “*there are things that are just between you and your doctor I think*” P26. Risks to breaking patient confidentiality were highlighted (B.Br.4).

#### 3.2.7 Decisional outcomes

Generally, participants believed at‐risk family members should be informed of the proband's LS diagnosis.

### 3.3 Theme C: navigating risk reducing interventions

#### 3.3.1 Theme

Participants felt navigating decisions related to risk‐reducing interventions were important throughout the siblings' LS diagnosis scenario.

#### 3.3.2 Initial considerations

Factors immediately considered were the proband's personal circumstance, gender, personal beliefs, and knowledge of interventions (Figure 1C). Participants reported difficulties in responding to the risk‐reducing surgery scenario due to being a different gender to the character (C.Br.1.RRS), and seriousness of procedure (C.Br.3.RRS).

#### 3.3.3 Barriers and facilitators

Physical implications such as menopause and its related implications (C.Br.2.RRS), and physical toll of surgery (C.Br.3.RRS) were considered barriers to undergoing risk reducing surgery. Psychological barriers were also identified (C.Br.4.RRS). Barriers towards annual colonoscopy surveillance were discomfort (C.Br.5.Cs) and risk of procedural complications (C.Br.6.Cs).

Minimising cancer risk was a key facilitator as participants viewed both interventions as proactive and preventative, “*Just the thought of catching it [cancer] early…without having it [risk reducing interventions] then you might catch it a lot later and the implications could be a lot worse*.” P41**.** Knowledge of interventions was also a facilitator to uptake (C.Fr.3.Cs) (C.Fr.4.RRS) “*when I read up on it [ovarian cancer], it was hard to diagnose…maybe because it is a harder one to diagnose…she should have them removed.”* P1. For risk‐reducing surgery, female proband age and family completeness were facilitators (C.Fr.1.RRS), (C.Fr.2.RRS).

#### 3.3.4 Positive reinforcers and enablers

The knowledge that these risk‐reducing interventions were the best available options to approaching a LS diagnosis was viewed as an enabler and positive reinforcer: “*if they'*re *at high risk and they've been told that that's the best…way to do it…[that] would be, a pretty good motivating factor*.” CP45 (C.Fr.5.Cs), (C.Fr.6.RRS).

#### 3.3.5 Negative reinforcers

One participant did not answer in favour of annual surveillance and expressed this may be too frequent given the invasiveness of the procedure (C.Br.7.Cs). Another participant expressed they may not uptake these risk‐reducing interventions if other options were available.

Only one participant decided against prophylactic surgery due to negative physical implications (C.Br.8.Cs).

#### 3.3.6 Subthemes

Cancer prevention was a key factor influencing the decision toward undergoing risk‐reducing interventions (C.Fr.6.RRS). Most participants felt benefits outweighed risks/negative implications. Participants recognized physical implications of surgery and possible complications deterring uptake, however all participants supported uptake of some form of risk‐reducing intervention, *“…if it means that they* can *pick up a cancer, or an abnormality precancerous and treat it then well, I want to be here for a long time.”* P26.

#### 3.3.7 Decisional outcome

Most participants (*n* = 21/23) answered in favour of both characters undergoing annual colonoscopy surveillance and in favour of Sarah undergoing risk‐reducing surgery (*n* = 22/23) (Table S3).

## 4 DISCUSSION

We found that individuals perceive wanting to know one's LS status, informing family members of their LS risk, and implications involved in risk‐reducing interventions as key decisions involved in the LS diagnostic testing pathway and navigating subsequent events. Participants weighed up decisions of potentially positive LS probands and subsequent family member considerations by exploring barriers and facilitators to the decisional outcome, and incorporated knowledge from evolving hypothetical scenarios to inform responses. At conclusion, some participants reported newly supportive personal attitudes towards genetic testing.

Findings suggest the evolving scenarios facilitated participants' capacity to make informed decisions. The interview itself may have acted as a knowledge‐/attitude‐influencing intervention, as support towards genetic testing increased pre‐post interview from 60‐85% (based on *n* = 20 participants), and overall by 91% (*n* = 21/23) by interview conclusion. The pre‐interview LS factsheet may have also been an influential educational component. These were unintended positive consequences of the scenario‐based interview and suggest the unfolding scenarios may have created an immersive and relatable learning environment, with interview questions enabling participants to apply their knowledge. Studies in the context of BRCA1/BRCA2 and prenatal genetic testing have illustrated benefits of using vignettes for facilitating decision‐making.^24^, ^25^ These findings suggest the scenarios used in this study could be valuable as an informational decision‐aid with potential application in LS or modification for other settings.

Recent public funding changes for LS genetic testing in Australia^4^ may progressively shift clinical responsibilities for diagnosing probands from genetic specialist services to oncologists or treating clinicians (known as ‘mainstreaming’ of genetic counselling and testing).^26^ Given challenges reported by non‐genetic counsellor healthcare professionals in delivering genetic counselling^27^ and encouraging probands to share information with relatives,^28^ these scenarios have potential to be further developed as a tool to support clinicians in enabling informed decision‐making.

Key considerations around LS genetic testing identified in this study align with findings from prior studies, including with familial cancer clinic attendees^29^ or those recently diagnosed with CRC.^30^ In our study, knowledge emerged as a fundamental facilitator in decision‐making processes across all three themes. Knowledge acted as a facilitator to aid decision‐making and reduce perceived negative enforcers or barriers around genetic testing.

We also found negative emotional experience (e.g., fear and anxiety) was a key barrier across all themes. Participants expressed concerns about financial implications associated with genetic testing and subsequent risk‐reducing interventions and insurance implications. These concerns are well documented.^17^, ^31^, ^32^ Our study occurred before protective genetic discrimination regulations were introduced. Public funding was also not available for LS testing at time of interview. Consistent with our findings, a previous study reported misconceptions among both patients and providers about potential high out‐of‐pocket costs associated with genetic testing as barrier to LS genetic referral.^33^ Whilst insurance reforms and new public funding may alleviate financial concerns, further efforts may be needed to ensure providers are aware of changes and that patients receive appropriate information to make informed choices about genetic referral and testing.

Most participants supported recommended risk‐reducing interventions, but were also concerned about potential associated side‐effects and discomforts. A recent Australian nation‐wide audit of risk management in LS carriers across 12 familial cancer clinics reported main reasons for clinical recommendation non‐adherence, which included feeling uncomfortable and/or disruption associated with colonoscopy, and, for female LS carriers, younger age and family incompleteness.^34^ Vignettes described a male proband and female sibling. It would be interesting to explore whether switching genders would elicit alternative perspectives, given gender has been found to influence communication around LS.^35^

We recruited participants without cancer history. Given universal tumour testing for LS in all incident CRC cases is likely to occur in Australia, it is important to understand these individual's perspectives on LS genetic testing. However, Australian research has focused on high‐risk populations (individuals with dMMR tumours or confirmed LS) and is unlikely to capture testing pathways relating to systematic LS testing in all CRC cases.^10^, ^14^, ^15^, ^16^, ^17^, ^18^ Therefore, this study provides insight into potentially effective and feasible approaches to facilitating decision‐making regarding LS genetic testing and related events, when systematic LS testing is introduced. Decision‐making processes are likely not limited to LS, therefore it is possible for these findings to be expanded across other hereditary conditions with similar potential actionability. We have developed a microsimulation modelling platform *‘Policy1‐Lynch’* to evaluate the predicted impact, benefits, harms and cost‐effectiveness of various testing and risk management strategies for LS.^2^ Results from this study can be used as a range of parameter inputs to ‘*Policy1‐Lynch’* to investigate impacts of likely testing uptake and test result sharing rates on the cost‐effectiveness of systematic LS testing.

### 4.1 Study limitations

This study did not specifically investigate decisions of patients with an unconfirmed MSI status CRC diagnosis. Should Australia adopt universal genetic testing,^4^ our findings may still be relevant to these patients.

Lack of participant demographic information limited sub‐group analysis; however, our preliminary findings can be used as a foundation for future investigation towards specific differences in views between demographic groups within a larger sample. Most participants (18/23) were recruited from a cancer fundraising event and a suboptimal response rate led to additional convenience sampling. Therefore, views may not represent the broader population due to small sample size as well as potential favourable attitudes towards cancer related issues. Despite this, a variety of views towards genetic testing and vignettes were expressed. To account for potential positivity bias to influence convenience sample responses, we also checked the thematic integrity without convenience sample data and found no compromise.

Interviews examined anticipated behaviours and intentions, but evidence for the extent to which vignette‐driven interviews can predict future real‐life healthcare decisions varies. For example, in breast cancer genetic testing, differences have emerged between hypothetical scenario and real‐life decision‐making.^36^ By contrast, in‐depth consideration involved with hypothetical scenarios^37^ and use of effective methodologies within vignette‐driven interviews can illustrate predictive relationships between hypothetical responses and future decisions.^38^ Valuable insights can also be gained by understanding motivations and thought‐processes underlying LS testing decisions when framed hypothetically.^39^ This study did not control for social‐desirability bias, that is, participants modifying responses according to perceived interviewer expectations.^40^ Future investigations may benefit from specific measures to minimize social‐desirability bias (e.g. intentional rapport building).^41^

### 4.2 Clinical implications

This study highlights challenges faced by individuals moving through the LS diagnostic pathway, and key factors impacting decision‐making about genetic testing, uptake of risk‐reducing measures, and information dissemination to at‐risk relatives. Results emphasise the healthcare professional's role in communicating with patients about LS and facilitating information dissemination to at‐risk relatives. Given attitude changes pre‐post interviews, these vignettes may have potential as interventions (targeting both healthcare professionals and patients) to support informed decision‐making towards LS testing, and to aid implementation of mainstreaming genetic testing.

## 5 CONCLUSIONS

Interviews conducted via well‐informed hypothetical scenarios may support decision‐making processes involved in LS testing. This may have broader applications to other hereditary and health conditions which involve complex decision‐making processes involving multiple considerations.

## CONFLICT OF INTEREST

KC is co‐principal investigator of an unrelated investigator‐initiated trial of cervical screening in Australia (Compass; ACTRN12613001207707 and NCT02328872), which is conducted and funded by the VCS Foundation (VCS), a government‐funded health promotion charity. The VCS Foundation received equipment and a funding contribution from Roche Molecular Systems and Ventana USA but neither KC nor her institution on her behalf receives direct funding from industry for this trial or any other project. Other authors declare no competing interests.

## AUTHOR CONTRIBUTION

Yoon‐Jung Kang, Emily Hogden, Natalie Taylor and Karen Canfell contributed to the study's conception and design. Yoon‐Jung Kang, Victoria Freeman, April Morrow and Natalie Taylor developed the interview schedule. Yoon‐Jung Kang, April Morrow and Victoria Freeman conducted telephone interviews. Gabriella Tiernan and Victoria Freeman analysed the data and interpreted results. Yoon‐Jung Kang, Gabriella Tiernan and Victoria Freeman drafted the manuscript. Natalie Taylor had overall responsibility for study conduct. All authors provided comment on the manuscript and approved the final manuscript.

## Supporting information

Supporting Information 1

## Data Availability

The data supporting the findings of this study are available in the supplementary material of this article.
