# Supplementary material for: What would I do? Perspectives on the factors underlying Lynch syndrome genetic testing and results sharing decisions for high‐risk colorectal cancer patients
Source: Psychooncology. 2021 Nov 8;31(4):587–96. doi: 10.1002/pon.5840 (PMC9298871; doi:10.1002/pon.5840)
Supplement: Supplementary file 1 — Supporting Information 1 [file PON-31-587-s001.docx]

**What would I do? Perspectives on the factors affecting decisions around Lynch syndrome genetic testing and results sharing for high-risk colorectal cancer patients**

**Supporting information**

Gabriella Tiernan^1*^, Victoria Freeman^1*^, April Morrow^1,2^, Emily Hogden^1^, Karen Canfell^1,2^, Yoon-Jung Kang^1^†, Natalie Taylor^1^**^,^**^3^†

^1^ The Daffodil Centre, The University of Sydney, a joint venture with Cancer Council NSW

^2^ Prince of Wales Clinical School, University of New South Wales, Sydney, New South Wales, Australia

^3^ School of Population Health, Faculty of Medicine, University of New South Wales, Sydney, New South Wales, Australia

* Joint first authors

† Joint senior authors

**CORRESPONDENCE:** Associate Professor Natalie Taylor

School of Population Health

Faculty of Medicine

Samuels Building

University of New South Wales, Sydney, NSW, 2052, Australia

Email: [natalie.taylor@unsw.edu.au](mailto:natalie.taylor@unsw.edu.au)

**SHORT TITLE: Perspectives on genetic testing for Lynch syndrome**

**Table of contents**

[1. Further information on Lynch Syndrome and systematic testing for Lynch Syndrome 3](#_Toc85708403)

[2. Lynch syndrome fact sheet provided to participants prior to interview 3](#_Toc85708404)

[3. Script for the telephone interview 4](#_Toc85708405)

[4. Table S1. The Consolidated criteria for reporting qualitative studies (COREQ) checklist 9](#_Toc85708406)

[5. Table S2. Detailed breakdown of barriers and facilitators within each theme ((A) Wanting to know of one’s LS status; (B) Sharing the results; and (C) Risk reducing interventions) and key quotes 14](#_Toc85708407)

[6. Table S3. Responses to related close-ended questions around hypothetical scenarios regarding LS diagnosis pathways and personal views on genetic testing: Initial and subsequent answers compared 16](#_Toc85708408)

[7. Table S4. Extended tables of quotes to support themes 18](#_Toc85708409)

[8. References 27](#_Toc85708410)

# Further information on Lynch Syndrome and systematic testing for Lynch Syndrome

Lynch Syndrome (LS) is caused by constitutional mutations affecting four DNA mismatch repair (MMR) genes and approximately 3% of all colorectal cancer (CRC) cases are due to LS with their tumours demonstrating this mismatch repair deficiency (dMMR)(1). However, an additional ~12% of sporadic CRC cases also demonstrate dMMR in their tumours due to the presence of the somatic BRAF V600E mutation and/or hypermethylation of the MLH1 promoter region (2, 3). Rapid genetic and genomic discoveries have shifted identifying LS carriers from clinical assessment based on family history to dMMR tumour testing, with the latter approach offering greater sensitivity and specificity. Accordingly, the most common practice for identifying LS carriers has become molecular dMMR tumour testing in CRC cases with an optional somatic mutation test to rule out non-LS dMMR tumours and sequential germline genetic testing to confirm LS(3). If patients are found to be mutation carriers (i.e. proband), their at-risk relatives have the option of undergoing predictive genetic testing to clarify their own cancer risks (5).

It has been reported that universal dMMR tumour testing for LS in all incident CRC cases (systematic testing) is found to be cost-effective (6, 7). As a result, systematic LS testing in all CRC cases is recommended in some developed countries such as the UK (8). In Australia, systematic LS testing in all incident colorectal and endometrial cancers is likely to be implemented in the near future given germline genetic testing for LS and other hereditary conditions associated with increased CRC risk is Medicare funded as of 1 May 2020 (9). Studies have also reported that the cost-effectiveness of a LS testing program in CRC cases were sensitive to uptake rates of genetic testing for both probands and their at-risk relatives and adherence to colonoscopic surveillance in confirmed LS carriers (6, 7, 10, 11).

# Lynch syndrome fact sheet provided to participants prior to interview

Bowel cancer is a common cancer and tends to occur by chance in those over 50 years of age. It is unusual for a person to have many family members that have been affected by bowel cancer, or to experience bowel cancer at a young age. In this case, it is more likely that the cancers in the person or the family could have a genetic cause, which can be passed from one generation in a family to the next. Lynch syndrome (LS) is a genetic condition where one of a group of four genes stops working which then gives a person a higher chance of getting bowel, womb or ovarian cancer.

To break it down a bit further, when we say that LS is a genetic condition, this means it involves our genes. Genes are the smallest unit of inheritance. We have genes to control our growth, development and physical characteristics. Genes come in pairs, one from each of our parents. For LS, both copies in a pair of genes are required to function to prevent the development of cancer. In families where LS is inherited in this way, bowel cancers are usually seen in each generation. When a parent with LS passes on their genes, their child will randomly receive either the parent’s working or non-working LS gene. Thus, each child will have a 50% chance of having LS.

The first step in finding out whether someone’s bowel cancer is likely to be caused by LS, is to look at a sample of tissue from their tumour. There are certain genetic features in tumours that indicate whether someone is likely to have LS. If the tumour testing shows that it is likely to be caused by LS, then a genetic test on their blood sample can be done. Prior to this genetic test, genetic counselling is offered to assess an individual’s personal and family medical history, interpret test results, and explain what these results mean for individuals and their relatives. Genetic counselling also involves explaining the medical management implications of a positive or a negative genetic test result, providing referrals to medical specialists, support resources, research opportunities, and addressing concerns about the privacy and confidentiality of personal genetic information.

While the genetic test for bowel cancer does not provide an answer for everyone, genetic counselling can provide some recommendations. In families where an LS gene is found or where there is a strong family history of cancer and no LS gene found, guidelines recommend that your immediate family members start having bowel cancer screening by colonoscopy every year. As LS can be associated with womb and ovarian cancer, guidelines also recommend that women consider surgery to remove certain reproductive organs after she has finished bearing children.

There are a number of decisions to make and events that patients need to encounter in relation to the process described above, which are likely to increase the risk of both physical and psychological distress, examples of which include:

- The cancer diagnosis and treatment
- Being informed of being at high risk (including understanding complex information about genetics)
- Making the decision to take the test
- Waiting for the test results
- Finding out test results are positive
- Informing family members of their risk (potentially siblings and own children)
- Undergoing annual colonoscopies – a very invasive and potentially harmful procedure
- Waiting for screening results to see whether or not a cancer has developed
- Deciding whether or not to have surgery to remove organs in order to reduce cancer risk
- Potential financial burden – e.g., in relation to life insurance, health insurance, mortgage insurance, etc.

For more information, please visit Lynch Syndrome Australia website (<https://lynchsyndrome.org.au/>).

# Script for the telephone interview

Hello. May I speak with XXX?/ is this XXX?/ HI XXX?

My name is XX – I work at Cancer Council NSW Research Division.

You might remember a colleague of mine contacted you earlier to schedule for a telephone interview regarding the perspective of genetic testing for Lynch syndrome. Is this a good time for you to continue?

[if yes] OK great. Before I begin the interview, I would like to let you know that you have the option to withdraw at any time during this interview and remind you that this telephone interview will be recorded for transcription purposes but will remain confidential.

[if no] That is perfectly fine and I can reschedule for another time that is convenient to you.

**Introduction**

Before I begin the interview, I just like to check whether you had a chance to read the LS fact sheet that you received by an e-mail.

Would you like me to go over the shorter version of the fact sheet before going through the hypothetical scenarios or you prefer to start the interview straight away?

OK great.

Firstly, I would like to ask you “what is your opinion about genetic testing?”

[Next, I will brief you with the following information. It will help you to gain a better understanding of the situations we describe below for the two people we talk about in our evolving hypothetical scenario.]

As summarised in the LS fact sheet, bowel cancer is common and usually occurs by chance. It is less common, however, for families to have multiple people affected by bowel cancer, especially at young ages. This raises the possibility of an underlying genetic cause. We have genes to control our growth, development and physical characteristics, and also genes that protect us from cancer. Our genes come in pairs, and we get one copy from each of our parents. Lynch syndrome (LS) is a genetic condition where a person has a faulty copy of one of four ‘cancer protection genes’, giving a higher risk of bowel, womb or ovarian cancer. When a person with LS has children, their child will randomly receive either the working or faulty copy, and thus have a 50% chance of having LS.

When a person has bowel cancer, screening tests are done on their tumour tissue to see if LS is likely. If the screening test shows that LS is likely, the person can undergo genetic testing, using a blood sample, to make the diagnosis of LS. Prior to having the genetic test, genetic counselling is offered to explain what the results might mean for the person, and for their relatives. The genetic counsellor will also discuss the medical management recommendations, arrange any specialist referrals, provide psychosocial support and address any privacy or insurance concerns. Genetic testing and counselling would not involve any out of pocket costs.

After a person is diagnosed with LS, their relatives can then undergo genetic testing to see whether they also have LS. For those who do, guidelines recommend bowel cancer screening by colonoscopy every year. As LS can also be associated with womb and ovarian cancer, women are advised to consider surgery to remove certain reproductive organs once her family is complete. These guidelines make it possible for LS cancers to be prevented or detected early.

There are a number of factors during this process which people may find distressing, such as:

- The cancer diagnosis and treatment
- Being informed of being at high risk (including understanding complex genetic information)
- Deciding to take the test and waiting for results
- Coping with a LS diagnosis
- Informing family members of their risk (including own children)
- Undergoing annual colonoscopies and waiting for screening results
- Deciding whether or not to have surgery to reduce cancer risk
- Potential financial burden – e.g. life insurance

So, now I am going to provide you with an evolving scenario involving a brother and sister, James and Sarah. There are 3-4 parts to the scenario with different aspects to consider. Please think about the questions asked from the perspective of James and Sarah.

**Hypothetical scenario part 1.**

| **Hypothetical scenario** | **Interview questions** |
| --- | --- |
| James, a 38-year-old teacher, has been diagnosed with bowel cancer. During his treatment the doctors explain that his tumour was tested, and results show that his cancer may be linked to a genetic condition called Lynch syndrome. Some of his aunts and uncles on his mother’s side of the family had bowel cancer when they were in their sixties. Genetic testing is required to confirm if James has the condition. Lynch syndrome increases the chances of more cancers, such as a second bowel cancer or, for women, cancer in the uterus or ovaries. | 1. [Thinking about some of the factors I described earlier] What do you think James would consider before making the decision to undergo genetic testing?  2. A bit more about your thoughts on Q.1  a) Can you tell me a bit more about your thoughts on that?  b) After considering these factors, do you think James should uptake genetic testing? (Yes/No)   1. Could you tell me the reasons why and why not?   3. Results of genetic testing could have an impact on other family members, what might James consider in this context if he decided to have genetic testing? |

**Hypothetical scenario part 2.**

| **Hypothetical scenario** | **Interview questions** |
| --- | --- |
| James has decided to undergo genetic testing and has been informed that he carries a gene that causes Lynch Syndrome. His doctor explains that his children, brothers and sisters will have a 50% chance of having the same gene and that this was most likely passed on from his parents. Also, the other cancers that happened in his aunts and uncles could be related to Lynch syndrome. | 1. He just received this information after his cancer diagnosis. How do you think this information would affect James?  2. Because James’ immediate family members are also at risk of having Lynch syndrome, what do you think James might consider when making a decision about whether to share his genetic information?  3. Do you think he should share his test results with family members? (Yes/No)  a) Can you tell me a bit more about your thoughts on that?  b) Could you tell me the reasons why he should and should not?  4. How and when do you think he should share this information with his family?  a) Do you think there would be motivating factors or challenges to sharing this information?  5. In your opinion does James have the responsibility to share the genetic result with his family?  6. If James decided not to tell them, what responsibility do you think the healthcare professional has in relation to informing family members? |

**Hypothetical scenario part 3**

| **Hypothetical scenario** | **Interview questions** |
| --- | --- |
| James has a sister, Sarah, who is 45 years of age and has two teen-age children. James has decided to tell Sarah that his cancer was linked to Lynch syndrome, meaning Sarah also has a 50% chance of having Lynch syndrome, and this increases her risk for cancers such as bowel, uterus or ovary. James suggested to Sarah that she might want to meet with a genetic counsellor and have genetic testing to confirm whether she also has Lynch syndrome or not. | 1. What factors do you think Sarah might consider before making the decision to undergo genetic testing?  2. A bit more about your thoughts on Q.1  a) Can you tell me a bit more about your thoughts on that?  b) After considering these factors, do you think Sarah should uptake genetic testing? (Yes/No).   1. Could you tell me the reasons why and why not?   3. Before she makes a decision to undertake genetic testing or not, do you think Sarah should discuss having a genetic test with her children and family? (Yes/No)   1. What might influence her decisions? |

**Hypothetical scenario part 4**

| **Hypothetical scenario** | **Interview questions** |
| --- | --- |
| Sarah decided to have genetic testing and found that she also has Lynch syndrome. James and Sarah have several ways to reduce their cancer risks. For example, regular colonoscopy will help to find a bowel cancer at an earlier stage so less aggressive treatment will be needed and the chance of survival will be better. A colonoscopy is a visual inspection of the whole length of the large bowel using an instrument called colonoscope. There is a very small chance that serious complications might occur such as damage to the bowel or bleeding during colonoscopy. For Sarah, she also has the option to undergo a risk-reducing surgery to remove her ovaries and womb, now that she has finished having her children. | 1. Do you think James and Sarah should have annual colonoscopies?  2. Do you think there would be motivating factors or challenges in undergoing annual colonoscopies?  3. What are your thoughts about Sarah removing her reproductive organs to prevent cancer?  a) Do you think Sarah should undergo this risk-reducing surgery? (Yes/No)  4. How do you think James would feel knowing that his sister might have to undergo surgery to remove her reproductive organs?  5. We discussed about sharing his genetic information with his family members, now that he knows that his sister might have to undergo surgery, would this knowledge affect his decision to share his genetic information?  6. Do you think this knowledge would affect his decision to share his genetic information with his family members? (Yes/No)  a) Could you tell me the reasons why and why not? |

**In conclusion**

Before we conclude the interview, I have a few more questions and would like you to answer them from your own perspective (i.e., not from the perspective of James or Sarah).

Now that we have explored a number of issues relating to genetic testing in cancer, I am wondering:

1. How has your perspective of genetic testing in cancer changed since the start of the interview?
2. Is there anything else that would encourage you or deter you from doing genetic testing?
3. Other than what we’ve already talked about, is there anything else about the use of genetic testing in bowel cancer that worries you?
4. Are there circumstances where you would absolutely not test? I would like to mention that under Australian law, a patient who is made aware of the risk of a genetic condition may choose to advise relatives themselves, or they may consent to their doctor informing relatives on their behalf. However, where consent is not given, the Privacy Act does allow a doctor to use or disclose a patient’s genetic information to blood relatives if a number of conditions are met. This can only happen if the doctor reasonably believes that the use or disclosure will lessen or prevent a serious threat to the life, health or safety of a genetic relative.

Thank you for taking part in this interview. We would like to understand your own feelings about this interview rather than from the perspective of James and Sarah.

1. How has reading the scenarios or answering the questions made you feel?
2. Has taking part in this interview made you think differently about the health of you or your family? If so, can you please tell me why.

I have no further questions today.

Before we finish, do you have any further questions you’d like to ask or points you’d like to make?

Thank you for taking part in this interview.

# Table S1. The Consolidated criteria for reporting qualitative studies (COREQ) checklist

The Consolidated criteria for reporting qualitative studies (COREQ) checklist(4)

| **Characteristics (in each domain)** | **Covered in article** | **Response** |
| --- | --- | --- |
| **Domain 1. Research team and reflexivity** | | |
| **Personal Characteristics** | | |
| 1. Interviewer/facilitator: Which author/s conducted the interview or focus group? | YES | AM, YJK and VF conducted interviews |
| 2. Credentials: What were the researcher’s credentials? E.g. PhD, MD | YES | Detailed in manuscript after the paper title |
| 3. Occupation: What was their occupation at the time of the study? | YES | Interviewers: Certified Genetic Councillor (AM), Post Doctoral Research Fellow (YJK), Systematic Reviewer (VF)  Coders: Systematic Reviewer (VF), Research Assistant (GT)  Supervising researcher: Senior Research Fellow (NT) |
| 4. Gender: Was the researcher male or female? | YES | AM, GT, YJK, VF, NT: Female |
| 5. Experience and training: What experience or training did the researcher have? | YES | - One interviewer (AM) has 5 years of experience as a certified genetic counsellor, which involves provision of information and psychosocial support to patients with hereditary cancer syndromes; also has experience conducting research interviews and undertaking qualitative analyses. - Two interviewers (YJK, VF) underwent the “Accidental counsellor” training course conducted by trained Cancer Council NSW support officers in preparation for interviews. This training course aimed to teach communication skills to assist in communicating effectively in those unexpected times when faced with a person's emotional reaction to cancer and its treatment. - One of the interviewers and coders (VF) has five years of experience in research, specialising in research methodology and also including protocol design and data analysis. - One of the coders (VF) underwent professional training for NVIVO data analysis software and imparted learnings onto the second coder (GT). - Senior researcher (NT) has 15 years of research experience in mixed methods research, including designing, conducting and analysing qualitative interviews, including inductive and deductive analysis, and triangulation of results. |
| **Relationship with participants** | | |
| 6. Relationship established: Was a relationship established prior to study commencement? | YES | Initial participants were recruited from Cancer Council NSW’s Relay For Life event where the researchers met participants for the first time.  The convenience sample consisted of friends and family of the interviewers (partner and mother of AM, three friends of VF). Due to the nature of relationships with interviewers among those in the convenience sample, it is possible that certain responses to personal questions may have been influenced by the relationship to the interviewers (e.g. the amount of openness that the participant responded).However, convenience sampled participants were aware that researchers were representing CCNSW and that any information collected in the interview was recorded and used for research purposes which may have mitigated providing overly open responses. |
| 7. Participant knowledge of the interviewer: What did the participants know about the researcher? e.g. personal goals, reasons for doing the research | YES | The purposive participants did not know the researchers personally prior or during research conduct. The convenience sample consisted of family or friends of two of the researchers (AM, VF). All participants were provided information about the context of the research, research goals, how the results will be used and why their input was important within the Participation Information and Consent form. Participants were encouraged to ask any further questions about the study at any point during the recruitment, interview or study period. |
| 8. Interviewer characteristics: What characteristics were reported about the interviewer/facilitator? e.g. Bias, assumptions, reasons and interests in the research topic. | YES | At the time of study conduct, YJK worked as a Post Doctoral researcher in the Lynch Syndrome research team at Cancer Council NSW led by NT.  AM has 5 years of experience as a certified genetic counsellor and at the time of study conduct was conducting a PhD within the Lynch Syndrome research team.  VF has a background in psychology and at the time of study conduct was working as a Systematic Reviewer separately from the researchers in the Lynch Syndrome team.  Participants were made aware in the Participant Information and Consent form that the Lynch Syndrome team are interested in gathering participant perspectives in this study to inform a cost-effectiveness evaluation of screening for LS in LS-related cancers. It may also be used to inform another CCNSW project aiming to improve genetic testing referral rates for bowel cancer patients. The results may also inform public policy for cancer prevention and management.  All researchers have an interest in LS and are supportive of genetic counselling, genetic testing and management of cancer risk. All researchers acknowledge that patients and members of the general public may not feel the same and are conducting this research to understand these views.  All researchers had no intent to elicit particular responses or interpreting the data in a way that supports any research agendas. |
|  | | |
| **Domain 2: Study design** | | |
| **Theoretical framework** | | |
| 9. Methodological orientation and Theory: What methodological orientation was stated to underpin the study? e.g. grounded theory, discourse analysis, ethnography, phenomenology, content analysis. | YES | An inductive thematic analysis approach was taken to analyse the dataset based on Braun and Clarke 2006 (12) |
| **Participant selection** | | |
| 10. Sampling: How were participants selected? e.g. purposive, convenience, consecutive, snowball | YES | Eighteen participants were recruited from Cancer Council’s NSW’s Relay For Life event and five participants comprised a convenience sample. The decision to recruit at a charity event was a test of feasibility for this type of recruitment strategy. |
| 11. Method of approach: How were participants approached? e.g. face-to-face, telephone, mail, email | YES | All participants registered face-to-face and the consent form was signed in person, so that researchers could answer any further questions. Upon agreement to participate, purposive participants were sent a confirmation of their interview time along with an email containing the same Lynch Syndrome fact sheet and hypothetical scenarios that they were provided in the Participant Information and Consent form. All convenience sample participants were physically handed their Lynch Syndrome fact sheet and hypothetical scenarios as provided in the Participant Information and Consent form. |
| 12. Sample size: How many participants were in the study? | YES | N = 23 (18 purposive, 5 convenience) |
| 13. Non-participation: How many people refused to participate or dropped out? Reasons? | YES | Forty-four individuals registered their interest at ‘Relay for Life’ and completed a written consent form to participate in the study. Of these, 26 individuals did not proceed further due to declining (n=3), no answer to multiple call-backs, number was disconnected or incorrect (n=23). This resulted in 18 remaining participants that underwent telephone interviews. All approached convenience sample participants (n=5) consented to participation in the study. |
| **Setting** | | |
| 14. Setting of data collection: Where was the data collected? e.g. home, clinic, workplace | YES | Interviews were conducted via one-on-one telephone interviews. Interviewers called participants from a private room at Cancer Council NSW. In the case of some convenience samples, interviews were held in-person at the home of the participant. In order to simulate the absence of non-verbal cues over telephone, the interviewer and participant faced away from each other during the in-person interview. |
| 15. Presence of non-participants: Was anyone else present besides the participants and researchers? | YES | No one else was present other than the participant and researcher conducting the interview. |
| 16. Description of sample: What are the important characteristics of the sample? e.g. demographic data, date | YES | Attendees of the ‘Relay for Life’ event were members of the local community (Sutherland Shire) or individuals with a personal connection to cancer (i.e. were cancer survivors or support people). |
| **Data collection** | | |
| 17. Interview guide: Were questions, prompts, guides provided by the authors? Was it pilot tested? | YES | The interviews were semi-structured in nature, consisting of both closed-ended and open-ended questions. The interview script and questions were pilot tested and refined based on feedback from three practice participants from Cancer Council NSW’s Research Division. Each interviewer conducted the interview based on the same questions that were finalised from the pilot test, however due to the semi-structured nature of the interview, some questions were modified or omitted to support conversation flow. |
| 18. Repeat interviews: Were repeat interviews carried out? If yes, how many? | N/A | None |
| 19. Audio/visual recording: Did the research use audio or visual recording to collect the data? | YES | All interviews were audio recorded on a Dictaphone and transcribed verbatim by a third- party transcription service. |
| 20. Field notes: Were field notes made during and/or after the interview or focus group? | N/A | No field notes were taken. |
| 21. Duration: What was the duration of the interviews or focus group? | YES | Each interview lasted around 30-45 minutes. |
| 22. Data saturation: Was data saturation discussed? | YES | It was agreed by the study researchers that recruitment could stop earlier if saturation were reached i.e. no new information or perspectives gained from additional participants and in the event that there were not enough participants initially recruited. |
| 23. Transcripts returned: Were transcripts returned to participants for comment and/or correction? | N/A | N/A |
|  | | |
| **Domain 3: Analysis and findings** | | |
| **Data analysis** | | |
| 24. Number of data coders: How many data coders coded the data? | YES | Data was independently coded by two data coders who were blind to the other’s coding (VF, GT) |
| 25. Description of the coding tree: Did authors provide a description of the coding tree? |  | Themes, subthemes and a sample of corresponding quotes are provided in the Supporting table 2 to give readers an understanding of the development of themes and subthemes. Themes, subthemes and a sample of corresponding quotes are provided in the supplementary file Table 4 to give readers an understanding of the development of themes and subthemes.  Coding was completed by systematic and independent coding by both reviewers of all transcripts. Description of the coding process, overarching theme, theme, and subtheme development are provided in the data analysis section of the methods. |
| 26. Derivation of themes: Were themes identified in advance or derived from the data? |  | Themes were derived from the data as per inductive thematic analysis methodology. |
| 27. Software: What software, if applicable, was used to manage the data? |  | Qualitative data coding was undertaken in NVivo 12 Plus software (QSR International Pty Ltd 2018)  Theme and subtheme development with quotes as well as recording of quantitative data was undertaken in Microsoft Excel. Interviews were transcribed in Microsoft Word. |
| 28. Participant checking: Did participants provide feedback on the findings? |  | As noted in the Participant Information and Consent form, participants were provided a summary of the results when the study was complete but feedback was not explicitly requested. |
| **Reporting** | | |
| 29. Quotations presented: Were participant quotations presented to illustrate the themes / findings? Was each quotation identified? e.g. participant number |  | Quotations were presented as frequently as possible both within the body of the text and in a separate table within the manuscript and also within the supplementary materials to illustrate themes or subthemes discussed. Pertinent quotes to each theme and subtheme are provided in the main manuscript (Table 3) and in Supplementary file (Table 4) |
| 30. Data and findings consistent: Was there consistency between the data presented and the findings? |  | One of the researchers (AM) was deliberately blinded to the themes and subthemes whilst reading all of the transcripts and then reviewed the thematic table and decision pathway diagrams. This researcher then met with the two coding researchers (VF, GT) to discuss similarities and discordances in data and findings. In the event that the thematic table and decision pathway maps did not accurately reflect the dataset based on reviewer feedback, coding was reviewed until an apt thematic table and decision pathway maps were produced. This process ensured consistency between the data presented and findings reported.  During write-up of the analysis, notes taken during decision-making were closely referred to so that rationale behind decisions could be reported and to ensure accuracy during further revision of themes and subthemes |
| 31. Clarity of major themes: Were major themes clearly presented in the findings? |  | Major themes are listed clearly in table 3 and Figure 1 and under clear headings in text. Subthemes are also clearly outlined in the results as well as the decisional pathway diagrams and the extended table of quotes contained within the supplementary material. |
| 32. Clarity of minor themes: Is there a description of diverse cases or discussion of minor themes? |  | Subthemes are clearly listed under major themes with their titles in bold font. Any instances where there were differences of opinions or opinions voiced by a minority but were thought to be important issues (after discussion by study researchers), these were included and reported. |

#

# Table S2. Detailed breakdown of barriers and facilitators within each theme ((A) Wanting to know of one’s LS status; (B) Sharing the results; and (C) Risk reducing interventions) and key quotes

| **Barrier or Facilitator** | **Key Quotes within each Theme (subthemes)**  **(Index number, Qualifier*, Quote, Participant number)** |
| --- | --- |
| ***Theme A: Wanting to know of one’s LS status (Subthemes: Knowledge as power, Fear of testing positive, Personal Beliefs)*** | |
| Fr | 1. *“I’d probably want to know because I’ve got three children I would want to tell them so they could find and in the next generation after that.” P1 (A.Fr.1)* 2. *“I’d rather know because that way then prevention is better than cure sort of thing you know if you know you’ve got it well then you can take steps” P8 (A.Fr.2)* 3. *“knowledge is power … you should know so that way you’re able to monitor… if you get things early enough that you do have a higher chance of survival and or cure.” P16 (A.Fr.3)* 4. *“once he’s sort of been given an adequate amount of information about what it is that they’re doing and the reasons why that genetic testing would be beneficial or could be beneficial to him, I don’t see why he wouldn’t, give it a try” CP45 (A.Fr.4)* |
| Br | 1. *“there is a fifty percent chance that you could have it …even if you do have it, it doesn’t definitely mean that you’re going to get cancer so, there’s some people who would take that and go I’ll take the risk…if it means they don’t have to undergo all of those procedures now.” P22 (A.Br.1)* 2. *“so it’s just about making lifestyle changes if necessary” P7 (A.Br.2)* 3. *“coming across that knowledge might make someone else so deeply unhappy that, that they hurt themselves” CP45 (A.Br.3)* 4. *“if they [health professionals] don’t give him that information then he’s gonna get something from somewhere else and maybe that’s not accurate … it might misinform” P43 (A.Br.4)* 5. “*they say: ‘no you’ve got this fifty percent chance so we’re not covering you for any cancer treatment’ and it could be completely unrelated cancers skin cancer or something… yeah the ethics of insurance companies would concern me*.” *P1* *(A.Br.5)* |
| ***Theme B: Informing family about LS (Subthemes: Responsibility, support, privacy)*** | |
| Fr | 1. *“the motivation would be that in the long run he could help them from going or stop them from going through what he’s going through at the moment.” P22 (B.Fr.1)* 2. *“the way to do it is to arrange you know to meet with the family maybe everyone face to face potentially with a genetic counsellor maybe a good way of doing it as well so that everyone can sort of ask questions you know and he has a bit of support when he’s delivering that information.” CP47 (B.Fr.2)* 3. *“he’s just the information passer-onerer but It empowers her to make that decision if he’s aware of it [lynch syndrome] that’s good because he needs to have as much information to support his sister as well” P5 (B.Fr.3)* 4. *“I think they need to support James as a priority yeah but… if it was me and I wasn’t speaking to my brother and that was the case I would probably still like to know… I think the health industry does have a responsibility in some way” P29 (B.Fr.4)* 5. *“in the long run he could help them from going or stop them from going through what he’s going through at the moment.” P22 (B.Fr.5)* |
| Br | 1. *“you put the fear into, you know, people that you love and you know you’re making them face reality on something that they probably hadn’t even thought about” P16 (B.Br.1)* 2. *“some people could react poorly in terms of increased stress because they know that this is in the family blood line so obviously anxiety and depression could become involved” P5 (B.Br.2)* 3. *“just the whole emotion of him going through it himself and then also having to tell them that” P9 (B.Br.3)* 4. *“if you start whittling down…patient’s confidentiality, where does it stop – you know – can an abusive husband go to the doctor and find out about stuff about his wife?” P1 (B.Br.4)* |
| ***Theme C: Navigating risk reducing interventions (Subthemes: Prevention of cancer, physical implications, benefit outweighs risk)*** | |
| Fr | 1. *RRS. ““Uh I think I would do it especially if she has had children I think that would be a no-brainer” P29 (C.Fr.1.RRS)* 2. *RRS:* *“I’d just say ‘Right, get rid of my uterus and ovaries and that’s fine, because I’m forty five and I’m done with having kids’” P26 (C.Fr.2.RRS)* 3. *Cs: “I’ve had colonoscopies myself and I’ve never really found it to be risky or a problem at all” P19 (C.Fr.3.Cs)* 4. *RRS. “I’d probably do it because ovarian cancer is one of the hardest cancers to detect… once they have been diagnosed it’s usually too late.” P7 (C.Fr.4.RRS)* 5. *Cs: “if it means that they can pick up a cancer, or an abnormality precancerous and treat it then well, I want to be here for a long time.” P26 (C.Fr.5.Cs)* 6. *RRS:* *“…definitely if I was her I would undergo that procedure to stop or to lessen the risk of cancer in the future” P22 (C.Fr.6.RRS)* |
| Br | 1. *RRS. “It’s probably a bit more difficult as a male… it’s hard to say being a male when it’s in relation to a female.” P41 (C.Br.1.RRS)* 2. *RRS. “obviously if she’s had her ovaries removed it would bring on like a premature menopause so she might have to have hormone replacement therapy and things like that.” CP47 (C.Br.2.RRS)* 3. *RRS. “…it would take a long time to come to that decision really because it would be big surgery with a lot of recovery” P22* *(C.Br****.3****.RRS)* 4. *RRS. “it depends on…your attitude to your reproductive self.” P1 (C.Br.4.RRS)* 5. *Cs. “I think they’re uncomfortable aren’t they? Don’t you have to take those drinks?” P17 (C.Br.5.Cs)* 6. *Cs. “Yeah definitely the risks of a colonoscopy like you said with the bleeding and everything like that it would be challenging.” P22 (C.Br.6.Cs)* 7. “I might be tempted not to have it annually but maybe every two or three years because of the invasiveness of the procedure” *CP48* *(C.Br.7.Cs)* 8. *RRS. “She’s going at 45, she’ll go through menopause if she does that [prophylactic surgery] and that’s got other risks afterwards for osteoporosis things like that… I think she’d have to look at exactly what the risks are for those cancers to make those decisions”* *P42 (C.Br.8.Cs)* |

Br = Barrier; Fr = Facilitator; RRS = Risk Reducing Surgery; Cs = Colonoscopy.

*Qualifier: for Theme C, a qualifier code is assigned to the quote to indicate what type of intervention the quote refers to.

Note: Participant numbers which are preceded with “C” indicate that this is a convenience sampled participant. Extended quotes may be found in Supporting information 7.

# Table S3. Responses to related close-ended questions around hypothetical scenarios regarding LS diagnosis pathways and personal views on genetic testing: Initial and subsequent answers compared

| **Topic (initial question I and subsequent question S)** | |
| --- | --- |
| **Response to Initial question (I)**  **(No. of respondents/total)** | **Response to subsequent question (S)**  **(No. of respondents/total)** |
| **Hypothetical scenario: Genetic testing uptake**  **I1. Should James uptake genetic testing? (Hypothetical scenario part 1, question 2b)**  **S1. Should Sarah uptake genetic testing? (Hypothetical scenario part 3, question 2b)** | |
| Yes (22/23) | Yes (22/22) |
| Conditional (1/23) | No (1/1) |
| **Hypothetical scenario: Result dissemination**  **I2. Should James share genetic test results with family member? (Hypothetical scenario part 2, question 3)**  **S2. Should Sarah share genetic testing plans with family members? (Hypothetical scenario part 3, question 3)** | |
| Yes (23/23) | Yes (partner and children) (9/23) |
|  | Yes (partner only) (6/23) |
|  | No (6/23) |
|  | Unsure (1/23) |
|  | Missing data (1/23) |
| **Hypothetical scenario: Responsibility to share genetic results**  **I3. Is James responsible for sharing genetic test results with family? (Hypothetical scenario part 2, question 5)**  **S3. Is health professional responsible for disclosing genetic test results with proband's family when proband chooses not to share? (Hypothetical scenario part 2, question 6)** | |
| Yes (21/23) | Yes (6/21) |
|  | Unsure (leaning toward yes) (3/21) |
|  | Unsure (leaning toward no) (1/21) |
|  | No (9/21) |
|  | Missing data (2/21) |
| Unsure (leaning toward yes) (2/23) | Unsure (leaning toward yes) (2/2) |
| **Hypothetical scenario: Preventative measures**  **I4. Should James and Sarah undergo annual colonoscopic surveillance? (Hypothetical scenario part 4, question 1)**  **S4. Should Sarah undergo risk reducing surgery? (Hypothetical scenario part 4, question 3a)** | |
| Yes (21/23) | Yes (19/21) |
|  | Conditional (1/21) |
|  | No (1/21) |
| Conditional (1/23) | Yes (1/1) |
| No (1/23) | Yes (1/1) |
| **Personal view: Personal attitudes toward genetic testing**  **I5. Attitude toward genetic testing at beginning of interview**  **S5: Attitude toward genetic testing at end of interview** | |
| For (12/20) | For (10/12) |
|  | Conditional (1/12) |
|  | Unsure (1/12) |
| Conditional (4/20) | For (7/7) |
| Neutral (1/20) |  |
| Open minded (1/20) |  |
| Unsure (1/20) |  |
| Unsure (1/20) | Unsure (1/1) |
| Not specified (3) † | For (3/3) |

†Personal attitudes were collected from n=20 participants at the beginning and n = 23 participants at the end of the interview.

Note: This table shows the responses to categorical questions. Related questions (i.e. questions about the same topic) are grouped together and described as initial (I) and subsequent (S) responses. Responses are categorised and quantities under each category are shown; Conditional responses refer to “yes” responses given on the terms of certain conditions named by the participant.

# Table S4. Extended tables of quotes to support themes

| **Category** | **Key quote** |
| --- | --- |
| **Theme A: Wanting to know of one’s LS status (subthemes: knowledge is power, fear of testing positive, personal beliefs)** | |
| **Considerations** | |
| Personal Circumstance | 1. “…*having kids she wants to be alive as long as possible and as healthy as long as possible so one of her considerations I guess would be: ‘anything that puts me at risk um I’d like to know about’*” P42 2. *“[he should consider] what he wants to get out of life in the in the future… also if he’s got any offsprings yet”* P41 3. *“…lifestyle elements or of is, who he is as a person… perhaps even his political views or, you know his if he has any religious views so, there’s a multitude of things that might affect his, you know what he you know what him as an individual might do in that situation.”* CP45 |
| Level of Knowledge about Lynch Syndrome and genetic testing | 1. *“… maybe get him some genetic counselling I think that’s the other thing too is understanding genetics and how things get transferred and passed on”* P7 2. *“he needs to make sure that he’s getting the research [about LS and genetic testing] from professionals…”* P43 |
| **Facilitators** | |
| Reduce family’s cancer risk (including protect family) | 1. *“If I were personally at a risk factor of something and there was a genetic component I’d probably want to know because I’ve got three children I would want to tell them so they could find [risk] and in the next generation after that”* P1 2. “*He would be able to help his sister. If she’s got nothing at this stage to find out the possibility of having the Lynch syndrome he could save her”* P12 3. *“Well I’d want to know for my children, I’d want to know if I was going to be passing it onto my kids.”* P26 4. *“[genetic testing] would help prevent cancer in his children in the future”* CP47 5. *“Given that there’s a high chance of her having Lynch syndrome I think that, she still has a strong moral obligation to find out for the sake of her kids”* CP48 |
| Information & Knowledge about genetic testing | 1. *“I that they need to be almost provided with that information [about genetic testing]. I guess people will always search for more but as long as they’re provided with the information to begin with then the doctors are helping that person make the choice”* P43 2. *“Once he’s sort of been given an adequate amount of information about what it is that they’re doing and the reasons why genetic testing… could be beneficial to him, I don’t see why he wouldn’t give it a try”* CP45 3. Interviewer: *“So if you knew a bit more about Lynch Syndrome and genetic testing, then that would encourage you or maybe discourage you?”* Interviewee: *“Encourage”* P29 |
| Prevention/ proactiveness | 1. *“[genetic testing] gives you an insight of um, what could happen in the future and, hopefully prevent the cancer from appearing.”* CP49 2. *“Maybe to know is to prevent”* P12 3. *“Possibly preventing or limiting the intensity of the treatment that she may have to get if she is diagnosed with it because they’d be able to catch it earlier”* CP45 4. *“I feel agreeing with genetic testing that- some of the terrible cancers could be avoided”* P18 5. “*prevention is better than cure… if you know you’ve got it well then you can take steps”* P8 |
| Control over cancer outcomes/knowledge as power | 1. *“if genetic testing is going to help in any way then I would prefer to know than not know”* CP46 2. *“he’s only 38 years old if he can know that this seems to appear in his family at 60 years old he may change his life or… change that coming out later in his life.”* P18 3. *“I think you should be more informed I think then you can make those decisions… rather than worrying about something that may not be, it’s better to worry about something that you can either control…”* P43 4. *“I feel like knowledge is power. It puts the ball in your court. You’ve got the power to either do what you want to do to try and not get the cancer or you can choose to constantly be having certain testing done to get it early… I just think the earlier the detection the better your chances are of survival”* P9 5. *“I actually see it [genetic testing] as a positive and you know I’m of the opinion that, you know knowledge is power and that you should know so that way you’re able to monitor… if you get things early enough that you do have a higher chance of survival and or cure.”* P16 6. *“Knowledge is power… she can then be aware of signs symptoms, getting regular checks and being sort of knowledgeable about her circumstance and process that”* P5 7. “*Telling the person that they are at risk of it means that they have the knowledge and the opportunity to help minimise or prevent it.*” CP45 |
| ***Barriers*** | |
| Fear of testing positive | 1. *“Yeah it just I guess the impact of a diagnosis on other people and not everybody copes, not everybody wants to know.”* P1 2. *“I guess it’s the fear of knowing you know that probably stops me at the moment”* P17 3. *“Although with a positive result he may, you know feel, guilt that he’s passed it on to his children if they were to have it, so it’s really quite complicated in, in that regard”* CP47 |
| Unintended negative consequences to knowing genetic information (including mistrust in use of genetic data) | 1. *"Concern me that they say ‘No you’ve got this fifty percent chance so we’re not covering you for any cancer treatment,’ and it could be completely unrelated cancers… yeah the ethics of insurance companies would concern me* **…** [I’m concerned by] how unethical insurance companies are… when you do your own insurance there’s pre-existing conditions whether you’re going to be knocked back for cover” P1 2. *“If it was like a private medical company that perhaps, had nefarious leanings or… If I was distrustful in some way of the, of the people who are undergoing it”* CP45 3. *“I’m for it if information can be kept ah in, hands that are safe so don’t want it to be used for private corporations’ benefits”* CP46 |
| Psychological distress | 1. *“I would hate to live every minute thinking ‘Oh my god oh my god is that something nasty?’”* P26 2. *“I think the dilemma is a bit like the question of: Do you know what day you’re gonna die? Genetic testing to some degree might start people being concerned and over worrying about whether they’re gonna get this disease or not because they now know they’re at greater risk for example.”* P42 3. *“…kind of mental feeling… you know that they’re kind of waiting to get cancer because of a genetic problem”* CP47 4. *“I guess if that knowledge coming across that knowledge might make someone else so deeply unhappy that, that they hurt themselves”* CP45 |
| Financial implications | 1. *“… financially home mortgage stuff like that I think that could be a big thing.”* P26 2. *“I don’t want to have to say this, but probably if it was expensive it might deter me”* CP45 3. *“I guess the cost will also be a factor for sure. And that will be obviously more of a factor for those with less and less money in society and in our community.”* P42 |
| Personal beliefs (including value received) | 1. *“She might think it’s not worth it potentially you know if there’s a fifty percent chance. She might… like to live on the edge so to speak.”* CP45 2. *“There is a fifty percent chance that you could have it but there’s also a fifty percent chance that you couldn’t… even if you do have it it doesn’t definitely mean that you’re going to get cancer so, there’s some people who would take that and go ‘I’ll take the risk’ … if it means they don’t have to undergo all of those procedures now.”* P22 3. “*I think if James had the information about lifestyle and about things like that he could share that with her could impart that knowledge with her. So it’s just about making lifestyle changes if necessary*” P7 4. *“Some people would have religious beliefs that would make them not do that type of thing”* P12 |
| Lack of information and knowledge on genetic testing | 1. *“… if they don’t give him that information [about genetic testing] then he’s gonna get something from somewhere else and maybe that’s not accurate or it might misinform”* P43 2. *“I think it’d be easy for him to be dismissive of it [genetic testing] without proper um information or having someone with the adequate amount of knowledge to explain exactly what it is that they’re doing so, I can see him being a bit sceptical maybe.”* CP45 3. *“I guess just being informed of everything could affect me both ways… if I was given all the information about everything that could go right and everything that could go wrong um I think that would help sway me you know?”* P22 |
| **Theme B: Informing family about LS (subthemes: responsibility, support, privacy)** | |
| ***Considerations*** | |
| Age of family members | 1. *"I would probably would maybe tell my husband but beyond that I wouldn’t tell the children until they needed to be concerned”* P43 2. “*I was her I would just do it [genetic testing] and I would treat them as mature enough to take that information into account and you know, they should know that as early as possible.”* CP45 3. *“Okay um she’s got teenage kids um she needs to be around um she doesn’t necessarily need to tell the kids at this stage until she knows anything”* P1 4. *“The difference between telling a 13-year-old and telling a 19-year-old a 19-year-old will probably deal with it a lot better than a 13-year-old.”* P6 5. *“I don’t think it would be necessary to tell her kids before getting the test because if she doesn’t have it, it would make them worry over nothing best to get the test and if it comes back positive then tell her kids and any other immediate family she cares to”* CP48 |
| Mindfulness of family reactions | 1. *“…how they [proband’s family] react to him having the cancer to begin with… I would probably be sharing that part first and then seeing how the family reacts to telling them the diagnosis and then if they seem to be okay- not too devastated by it… then I’d go ahead and tell them about the genetic testing"* P9 |
| Closeness to family members | 1. *“I would speak to the family members that I am most, closest to… maybe my siblings and then think about talking to other people.”* CP45 2. “*… if she is married then I would speak to the husband I don’t know if I would tell my children straight away, if she’s single I wouldn’t necessarily tell my children that… if she is married then certainly I would think you would speak to your husband about it.”* P42 3. *“Well if it was me I would be sharing that information with my relevant family members who I feel would need to be tested"* P18 4. *Interviewer: “In your opinion, does James have the responsibility to share the genetic results with his family?” Interviewee: “I probably think yes but everybody’s different. Depends on their family I suppose"* P18 |
| Timing of disclosing genetic information | 1. *“…you’d be fairly devastated and I think that I would just do it [share genetic information] straight away.”* P26 2. *“…after he’s got his results and then he can show the family the results and that they need to be tested and probably the sooner the better.”* CP49 3. *“I would do it [disclose genetic information] before the treatment”* P32 4. *"I think that’s the very first thing he should do I mean he needs to protect his family…immediately"* P19 |
| **Facilitators** | |
| HCP to provide support with talking about genetic testing | 1. *“…because he’s had genetic testing, he would have been offered genetic counselling so I think that would be up to the counsellors to try and make him make the correct informed decision with regards to telling the family”* P12 2. *“… the doctors would have less responsibility of sharing the information with other members of the family but their responsibility would be more about convincing the patient to share that knowledge with the rest of them… It would be better if the patient was involved in it initially but if they don’t want to be involved in it and they don’t want to tell their family members then I think the family members do have a right to access information that could affect them.”* P22 3. *“I believe that they should tell them… if a health professional knew that they should make that information known”* P16 4. *“I think they need to support James as a priority but… if it was me and I wasn’t speaking to my brother and that was the case I would probably still like to know… I think the health industry does have a responsibility in some way.”* P29 5. *“…James might be the type of person who mightn’t be dealing with that situation very well himself so it might be better coming from the health professional… the health professional should tell the sister because if I was the sister I’d want to know”* P8 6. “*Probably be the way to do it is to arrange you know to meet with the family maybe everyone face-to-face potentially with a genetic counsellor… so that everyone can ask questions and he has a bit of support when he’s delivering that information.”* CP47 7. *“…there might be some way that um the other family members could be contacted maybe not mentioning the family member that has it but maybe just asking them to go and have some testing”* P19 8. *“but if he doesn’t want his family members to know then they should do their best to sort of um try and keep him anonymous essentially”* CP47 9. Interviewer: *“Who do you think will be the best person to support to give to provide this kind of support for them for distress or anxiety?”* Interviewee: *“Genetic counsellors”* CP46 |
| Belief that family need to know LS positive result | 1. *“you could be endangering their life by not telling them”* P8 2. *“I think you have an obligation to pass it on.”* P1 3. *“I believe that they should tell them… if a health professional knew that they should make that information known”* P16 4. *“I do think there is a duty for the doctor to disclose that to them.”* P22 5. *“I think the health industry does have a responsibility in some way.”* P29 |
| Proband’s feelings of responsibility | 1. *“… Because there’s quite a strong history of it, and there’s such a risk within his direct blood line I think there is a responsibility [for James to inform family members of his LS status]”* P5 2. *“I believe it's up to the person who's having the testing”* P6 3. *“To me you have a responsibility to share that… you could be endangering their life by not telling them”* P8 4. *“He needs to do it and other people might find it difficult to cope with the news that it’s the worst possible outcome but I think you have an obligation to pass it on.”* P1 |
| Supportive family bonds | 1. *“I think he would be upset for her but he would support her 100%”* P32 2. *“Because at the end of the day… they’re genetically tested for it [LS] anyway so they have to stick together emotionally”* P32 3. *“I think he’ll feel supportive of her and hopefully empathetic to the situation because he wouldn’t want his sister to be in that high risk so he may actually feel proud of her for it”* P5 4. *“they’re in it together he’s been diagnosed with it as well… I think that he’d be quite sympathetic and supportive”* CP49 5. *“I think in a situation like this they could be good support for one another… the gene came from their parents so it’s something that they both share and so they could share the situation”* P8 |
| Prevention of cancer in family members | 1. *“… seeing his sister’s prepared to act on that information [it] might motivate him as well to know that his kids and family will be entrusted to know that information too so probably improve the chances of him sharing [LS positive genetic information]”* CP46 2. *“[disclosing LS status is] an opportunity to be able to prevent future cancers in the family I would have thought would be a good thing particularly as well if people are thinking of having children*” CP47 3. *“the motivation would be that in the long run he could help them from going or stop them from going through what he’s going through at the moment.”* P22 4. *“…he would share that information… his family can also go ahead and have the same testing just so that again they can catch it quite early rather than you know letting the cancer progress.”* P16 5. *“… how would James feel later down the track if he found out that one of them did have that form [caused by LS] of cancer? If he was able to share that information with them earlier, that they could’ve caught it earlier”* P16 |
| Decisional Autonomy of family members | 1. *“he would want to tell people to give them the choice of what they want to do with that information they can ignore it if they choose to”* P22 2. *“I’m sure he’d feel upset but she can make a decision herself… People aren’t forced to do things”* P43 3. *“… Giving people information and then letting them make the choice of what they do with it is important.”* P7 |
| Proband taking time to process diagnosis | 1. *“… he could wait until he’s been through his treatment and then he could talk to family”* P5 2. *“Probably [he should share the LS diagnosis with family] after he’s undergone treatment I know treatment can stretch out for a while but maybe not immediately after his got the diagnosis maybe just let it settle in and let him settle into how his life’s going to change first and everyone get used to the idea of him having cancer first and then yeah, …after the treatment’s over or after he’s settled into it then to reveal that to his family.”* P22 |
| Long term benefit of informing family members | 1. *“I’d hope that he would look more in the long run rather than the short-term”* P9 2. *“It would be more harmful in the long run for him not to share them [the genetic test results]. He might protect their worry now but in the long run it wouldn’t protect them at all with such a high risk of developing cancers”* P22 |
| **Barriers** | |
| Privacy | 1. *“I think it has to be that he’s happy to divulge the information and if he’s not I think that’s something you [healthcare professionals] just can’t step into…there are things that are just between you and your doctor, I think”* P26 2. *“I don’t see why automatically the doctor should be able to say to the family member ‘Hey, James has got Lynch Syndrome’ as opposed to like I said Ebola or something that’s highly infectious”* P42 3. *“I don't believe that it's really anyone else's business… I don't think that's the right of anyone [to disclose LS information] else though except for the person who has it… the genetic tester shouldn't have the right to let other people know without consent”* P6 4. *“… ultimately if it’s not something like HIV and you’re running around sleeping with- infecting people… patient confidentiality has to rule”* P1 5. *“it’s that thin edge of the wedge… if you start whittling down patient’s confidentiality where does it stop? Can an abusive husband go to the doctor and find out about stuff about his wife?”* P1 |
| Distance from family members | 1. *“depending on if they live close by or not… if it’s something that you’ve got to tell somebody over the phone rather than face-to-face that could prove to be a little bit of a challenge”* P9 |
| Difficulty processing own diagnosis | 1. *“…and just the whole emotion of him going through it himself and then also having to tell them that”* P9 2. *“not immediately after his got the diagnosis… just let it settle in and let him settle into how his life’s going to change first and everyone get used to the idea of him having cancer first…after the treatment’s over or after he’s settled into it then to reveal that to his family*." P22 |
| Not responsible for disclosing genetic information | 1. *“… I think some people might want to know and they would feel that he’s obliged to tell them and other people just won’t want to know so they’ll think it’s his responsibility not to tell them.”* CP46 2. *“I think personally I think he would have a responsibility um, but it’s up to every person on what they see it as you know some people might see it as ‘I don’t have a responsibility to tell anyone this is information that was given to me’”* P22 |
| Causing worry | 1. *“you put the fear into people that you love and you’re making them face reality on something that they probably hadn’t even thought about…”* P16 2. *“Having them be upset of hearing the news and obviously potentially devastating to them if they actually have it as well and have to have organs removed yeah that would have quite an impact on his family…”* P19 3. *“Once you know you’ve got something you spend time worrying about it”* P42 4. *“…he might be worried that you know he might be worried about the psychological implications that might come from her going through these procedures”* CP45 5. *“…some people could react poorly in terms of increased stress because they know that this is in the family line in the family blood line so obviously anxiety and depression could become involved”* P5 |
| Fear of Negative consequences | 1. *“It also may negatively impact on them because they may think that they shouldn’t have children if they had the genetic abnormalities.”* CP47 2. *“[family members could] Overreact possibly, or have their outlook on life it might add another layer and complexity to their already challenged life.”* P7 3. *“shame in the sense of … they’re talking to their partner that it might it might make them feel that they could put their kids at risk with their partner”* P41 4. *“Other people could get very mad with him, very upset, not wanting to have the test think he’s crazy I’m sure there would be some I’m sure that in certain families it could backfire on him.”* P29 |
| **Theme C: Navigating risk reducing interventions (subthemes: prevention of cancer, physical implications, benefit outweighs risk)** | |
| **Contributing Factors** | |
| Gender | 1. RRS: *“It’s probably a bit more difficult as a male… I would think it would be a better thing to do for in the long-term um but then once again it’s hard to say being a male when it’s in relation to a female.”* P41 |
| Personal circumstance | 1. RRS: *“I think that’s [decision to undergo risk reducing surgery] a personal thing.”* P17 2. RRS: *“that’s another that’s a hugely personal decision to make um and something that I think that I don’t know if I’d be qualified to make that decision I’m definitely not qualified to make that decision on behalf of someone else”* CP45 3. RRS: *“I can answer that easily yes because that’s not happening to me but if I was put in that situation I’m not sure.”* P18 |
| **Facilitators** | |
| Completeness of family | 1. RRS: *“if there was, you know, a very high chance then you know she’d had her children I would say yeah- you know remove them.”* P16 2. RRS: *“I think I would do it especially if she has had children. I think that would be a no-brainer*.” P29 3. RRS: *“[If] I was her and I looked at my family and said you know this is, this is my family this is what it is um and I’m sure that she’s wholly contented and you know loves her family dearly so if it was me yeah I would, I would get the surgery done*” CP45 4. RRS: *“If it was me I would do that I’m finished having my children also, so I would do that”* P18 5. RRS: *“I guess since she’s past the point of wanting to have children and everything it would be less difficult than if she was younger”* P22 6. RRS: *“I’d just say ‘Right, get rid of my uterus and ovaries and that’s fine, because I’m forty five and I’m done with having kids’”* P26 |
| Manage risk of cancer (prevention, proactiveness, reducing cancer risk, extending life) | 1. Cs: *“Yeah absolutely I think it [colonoscopy] should just be involved when you have a syndrome or an illness or whatever it is um I guess it’s just like that just getting a regular check-up even if it’s once a year just having that regular check up to make sure everything’s okay and then you can have peace of mind for the next year really.”* P22 2. Cs: *“Certainly Sarah thinks she’s around the age where there might be- it’s prudent to have a colonoscopy anyway… especially since she’s got relatives who have had bowel cancer.”* P42 3. Cs: *“if it means that they can pick up a cancer, or an abnormality precancerous and treat it then well, I want to be here for a long time.”* P26 4. Cs: *“Just the thought of catching it early… knowing that you could get it earlier then without having it, then you might catch it a lot later and the implications could be a lot worse”* P41 5. Cs: *“if they can pick up any early stage cancers then like you said… hopefully that would mean that they’d be less- easier to treat”* P43 6. Cs: *“I guess bottom line: you can’t put your head in the sand… if you’ve got the opportunity to do it you’ve got to especially if you’re such high risk because you don’t know when stuff can move fast.”* P1   RRS: *“fifty percent’s pretty high… I would probably [undergo risk reducing surgery]…* *when I read up on it it was hard to diagnose um yeah maybe because it is a harder one to diagnose you should she should have them removed now… do I want a little cancer timebomb ticking?”* P1   1. RRS: *“definitely if I was her I would undergo that procedure to stop or to lessen the risk of cancer in the future”* P22 2. RRS: *“it sounds to me like it’s a very good way of drastically minimising the risk of cancer*” CP45 3. RRS: *“…my understanding is that screening- there aren’t particularly effective screening tools for those kinds of cancers [ovarian]”* CP47 4. RRS: *“I’d probably do it because ovarian cancer is one of the hardest cancers to detect its usually too late ones are, ones that have been diagnosed it’s usually too late.”* P7 5. RRS: *“Yes I think she should just so that she can live life a little bit longer with her family”* P32 6. Cs: *“… to make sure everything’s okay and then you can have peace of mind for the next year really.”* P22 7. Cs: *“you can’t put your head in the sand”* P1 |
| Knowledge of intervention (best available option) | 1. Cs*: “if they’re at high risk and they’ve been told that that’s you know the best, one of the most effective ways to do it albeit there being a very small risk [of complication] … I would hope that for them that would be, a pretty good motivating factor”* CP45 2. Cs: “*I’ve had colonoscopies myself and I’ve never really found it to be risky or a problem at all”* P19 3. RRS: *“I’d probably do it because ovarian cancer is one of the hardest cancers to detect its usually too late ones are, ones that have been diagnosed it’s usually too late.”* P7 4. RRS: Interviewee: *“I suppose there could be umm there could be difficulties in either one that you choose but I just think that it’s good that um you get to have more than one option.”* Interviewer: *“Okay. What if colonoscopy is the only option?”* Interviewee: *“Then I would do that then.”* P32 |
| **Barriers** | |
| Physical implications of intervention (discomfort, menopause, preparation process, risk of complications) | 1. RRS: *“it would be difficult… Obviously if she’s had her ovaries removed it would bring on like a premature menopause so she might have to have hormone replacement therapy and things like that”* CP47 2. RRS: *“Going into early menopause… it depends on how women deal with it some people have an okay time some don’t then your attitude to your reproductive self I guess”* P1 3. RRS: *“…it would take a long time to come to that decision really because it would be big surgery with a lot of recovery and affect her in quite a way”* P22 4. Cs: *“I think they’re uncomfortable aren’t they? Don’t you have to take those drinks?”* P17 5. Cs: *“The risks of a colonoscopy… with the bleeding and everything like that, it would be challenging. I know the prep for colonoscopies can be quite annoying sometimes and irritating to have to undergo* P22 6. Cs: *“I might be tempted not to have it annually but maybe every two or three years because of the invasiveness of the procedure”* CP48 7. Cs: *“I understand the preparation is a bit icky and yucky as well and unpleasant.”* P1 |
| Psychological implications (attitude toward female body) | 1. Cs: *“I suppose mentally there would be [challenges] because you wouldn’t know what would happen after the test”* P32 2. Cs: *“Yes surely emotionally and the worries of the possible negative outcome”* P29 3. Cs: *“…the challenge of having to wait for the test results for the colonoscopy to come back”* P22 4. RRS: *“if it was me, I’m really not sure what I would do… there are some women out there that think if you don’t have a uterus you’re not a woman- I don’t think like that- If for some reason you’ve had your uterus removed”* P12 5. RRS: *“that’s a big ask for some women”* P18 6. RRS: *“Going into early menopause… it depends on how women deal with it some people have an okay time some don’t then your attitude to your reproductive self I guess”* P1 |
| Financial considerations | 1. Cs: *“I’m a teacher for example so me to have a day off you know it impacts my income… so I don’t know what her work situation is- if she’s a single mum…”* P1 2. Cs: *“The challenges will be if there’s a financial out-of-pocket I guess…”* P12 |
| Consideration of other available options | 1. Cs: *“If there are any other ways to monitor then I would probably select or look at any other options instead of choosing that one straightaway.”* P32 2. RRS: *“Uhm I would probably wait to see what her results are through the colonoscopy first and make that decision once she had all her information but if there was, you know, a very high chance then you know she’d had her children I would say yeah- you know remove them”* P22 |
| Maintenance and frequency of colonoscopy procedure | 1. Cs: *“… the challenges of having… the discomfort or even time, so if it’s going to be a regular thing finding time to put these [colonoscopies] in place.”* P41 2. Cs: *“I might be tempted not to have it annually but maybe every two or three years because of the invasiveness of the procedure”* CP48 |

HCP = health care provider; LS = Lynch syndrome; RRS = Risk Reducing Surgery; Cs = Colonoscopy; “P” indicates the participant number of the participant who stated the quote, participant numbers which are preceded with “C” indicate that this is a convenience sampled participant

Note: for Theme C, a qualifier code is assigned to the quote to indicate what type of intervention the quote refers to;

# References

1. Hampel H, Frankel WL, Martin E, Arnold M, Khanduja K, Kuebler P, et al. Feasibility of screening for Lynch syndrome among patients with colorectal cancer. J Clin Oncol. 2008;26(35):5783-8.

2. Chang L, Chang M, Chang HM, Chang F. Microsatellite Instability: A Predictive Biomarker for Cancer Immunotherapy. Applied immunohistochemistry & molecular morphology : AIMM. 2018;26(2):e15-e21.

3. Lynch HT, de la Chapelle A. Hereditary colorectal cancer. NEJM. 2003;348(10):919-32.

4. Tong A, Sainsbury P, Craig J. Consolidated criteria for reporting qualitative research (COREQ): a 32-item checklist for interviews and focus groups. International journal for quality in health care. 2007;19(6):349-57.

5. eviQ (Cancer Treatments Online): Referral guidelines for colorectal cancer or polyposis risk assessment and consideration of genetic testing (ID: 657 v.6): Cancer Institute NSW; 2019 [Available from: https://www.eviq.org.au/cancer-genetics/referral-guidelines/657-referral-guidelines-for-colorectal-cancer-or-p.

6. Kang YJ, Killen J, Caruana M, Simms K, Taylor N, Frayling IM, et al. The predicted impact and cost-effectiveness of systematic testing of people with incident colorectal cancer for Lynch syndrome. The Medical journal of Australia. 2020;212(2):72-81.

7. Snowsill T, Coelho H, Huxley N, Jones-Hughes T, Briscoe S, Frayling IM, et al. Molecular testing for Lynch syndrome in people with colorectal cancer: systematic reviews and economic evaluation. Health Technology Assessment. 2017;21(51):1-238.

8. National Institute for Health and Care Excellence. Molecular testing strategies for Lynch syndrome in people with colorectal cancer (Diagnostic guidance, DG27) 2017 [Available from: https://www.nice.org.uk/guidance/dg27.

9. May 2020 News: Changes to the MBS May 2020: Australian Government. Department of Health; [Available from: http://www.mbsonline.gov.au/internet/mbsonline/publishing.nsf/Content/20200501-News.

10. Assasi N, Blackhouse G, Campbell K, Gaebel K, Hopkins R, Jegathisawaran J. DNA Mismatch Repair Deficiency Tumour Testing for Patients With Colorectal Cancer: A Health Technology Assessment [Internet]. Ottawa (ON): Canadian Agency for Drugs and Technologies in Health; 2016 Aug. (CADTH Optimal Use Report. No. 5.3b). Available from https://www.ncbi.nlm.nih.gov/books/NBK384771/

11. Snowsill T, Huxley N, Hoyle M, Jones-Hughes T, Coelho H, Cooper C, et al. A systematic review and economic evaluation of diagnostic strategies for Lynch syndrome. Health Technology Assessment. 2014;18(58):1-406.

12. Braun V, Clarke V. Using thematic analysis in psychology. Qualitative research in psychology. 2006;3(2):77-101.
